# Advantages of Intensive Dental Courses

**Published:** 1920-05

**Authors:** J. M. Hale

**Affiliations:** Mount Vernon, Ind.


### ADVANTAGES OF INTENSIVE DENTAL COURSES

BY J. M. HALE, D.D.S., MOUNT VERNON, IND.

Several years since it was my good fortune to take personal instruction in operative dentistry under the tutelage of Dr. W. G. Crandall, then of Spencer, Iowa. Dr. Crandall gave me an incentive to progress such as I never experienced before, and I owe him a great debt of gratitude. He also gave me my first insight into the benefits and operations of study clubs, and made it possible for me to visit some of the successful clubs of the Northwest, and observe their work at close range. From the knowledge and impetus gained from Dr. Crandall, the Woodbury Study Club of Indiana was organized through the humble efforts of Dr. W. G. Downs, of Evansville, Ind., and myself, and with Dr. Chas. E. Woodbury, of Iowa, as teacher and demonstrator. It has been in continuous and successful operation for about five years.

The need for such study clubs, as have been in operation in the Northwest for many years, is now apparent. I believe the first study club was organized in St. Paul over fifteen years ago under the leadership of Dr. E. K. Wedelstaedt. From it have gone forth such operators as Woodbury, Clack, Searle, James, Finn, Conzett, Crandall and others.

What is the purpose of these study clubs? To develop the dentist in practical knowledge. The graduate from the dental college is equipped with much theoretical knowledge of his profession, but has had little practical work. If his ambitions do not soar very high, he is satisfied to do what he can do best, and about as well as the other fellow in his neighborhood is doing. Thus, in all departments of his profession he is seeking the lines of least resistance.

He will not practice accurate cavity preparation. He will not make many goldfoil fillings, for instance, "as it takes too much time"; "is too hard on the patient"; "his people will not remunerate him for such dentistry," and so on *ad nauseam*. You have all heard these lame and senseless excuses. Why does he make these excuses? Because he is not proficient, and is seeking to justify himself by repeating these falsehoods until he finally believes them to be true. The same conditions are true in all branches of dentistry that are developing new ideas and technic.

Within very recent years we have seen the advent of the X-ray in dentistry, conductive or regional anesthesia, new ideas as to the systemic effects arising from pulpless teeth and pyorrhea, new developments in prosthesis, new methods of impression-taking and articulation, removable bridgework and scientific work in typal forms of teeth. Many men have been contributors to the knowledge of these subjects whose names have no mention in this paper, but to whom grateful recognition is due and tendered.

How many dentists learned these things in college? How many became proficient in a single branch mentioned? Why? Can you learn to do these things by listening to a paper read before a dental society, or by reading a description of a technical subject in mere reports? Some few geniuses may, but the masses never. No! You must do the actual work under the guidance of some one who is an expert and a teacher.

No lecturer, instructor, or demonstrator can go before his pupils and expound the principles upon which his subject rests, explain how this or that operation should be performed, instance model operations and operators, warn against the errors into which his pupils are liable to fall, and then go away imagining his work is done, and that his pupil is now, or under such training, likely ever to become an operator. In addition to careful teaching he must perfect the practice under his watchful eye and trained hands, and insist upon proper manipulation and technic.

If before your entrance into a study club of such scope and character as I have described, you imagine you are a real operator, you have a real awakening period of disillusionment before you.

Dentistry under such conceptions as are demanded now is no easy art, and the conscientious dentist of today and the future must ever work hard.

Summarizing, I would say some of the benefits of study club work are:

First—Personal instruction.

Second—Concentration of effort.

Third—Unity of purpose.

Fourth—Close contact, and intimate associations with our instructors.

Fifth—Close friendships formed through intimate acquaintance.

Sixth—Incentive to work on account of mental telepathy occasioned by a number of men thinking and working on the same thing at the same time.

Seventh—Enthusiasm and higher ideals obtained through intensive study.

Eighth—Instead of the usual methods of instruction where the instructor does the work and the class watches, with the study club methods the class makes the operations assisted by suggestions from the instructor.

To deny the need of intensive instruction in all branches of dentistry is but to controvert a glaringly obvious fact. There is a need for absolute change of technic in some of our operative and mechanical procedures, not only from a prophylactic viewpoint, but for constructive measures to preserve vitality of teeth as well.

Our aims and ambitions have been too low. The term ''get by'' should never be in any dentist's vocabulary. The truth as expressed by Dr. Woodbury that all that separates a dentist of the first rank from one of the second rank is attention to details, can not be too strongly impressed. No operation is good enough until it is perfect.

By way of contrast, reflect upon the standards advocated by Drs. Orton, Tinker and Maves in crown and bridgework, and bring to mind the almost entire absence of any standard in about ninety-nine per cent. of all such work we have ever seen. By way of consolation, but not of palliation, we may say this dereliction is not confined to the lower, nor even the middle strata of our profession, but is equally as conspicuous and flagrant in the so-called and would-be highbrows of the fraternity.

It is only too apparent that much of our crown and bridgework today covers a multitude of sins and microbes; is absolutely devoid of esthetics, inefficient, inexpressibly horrible, a menace instead of a benefit. It is a sad commentary upon our boasted professional advancement in the crown and bridge art, but a too glaring fact, that only in a very few cases do we see even creditable work. The majority of crowns and bridges are living or dead monuments, sacred to the memory of our ignorance, stupidity and inability, as Dr. Prime has well said.

To "right about face" means hard work, but work is the birthright of the human race—not degrading, but uplifting, and a decoration of honor.

The history of denture construction has been one of evolution. My first effort to advance in prosthesis was in trying to master the Snow system with face bow and Snow anatomical articulator. I had frequent access to the work of Drs. Prothero, Wilson and the late Grant Molyneaux, and derived much benefit therefrom. My great desire was always for a simple technic that this system afforded.

The first great change in impression technic from the standard plaster impression that it was my privilege to see was the technic worked out by Greene Bros., and afterward elaborated by Mr. Supplee. The first regular course in instruction I had the privilege of attending was a course of one week, given by Mr. Supplee. My early experience with Hall methods always kept in front of me the complication of technic from a teachable viewpoint. These men did the best they could in a course of such short duration, and disseminated much knowledge, but, of course, the student could not possibly absorb or retain the technic in such short acquaintance of the subject, and no opportunity was afforded of doing any work under the guiding hand of these instructors.

That, in my opinion, is the reason for the comparative failure of these very short courses, and is the reason for my plea to the universities to give proper extension courses of a few weeks' duration where the students may have intensive instruction and the opportunity of actually performing the work taught under the guidance of a proper instructor.

I recently had opportunity to observe some work in full denture construction of the students in Indiana Dental College with methods taught by Dr. M. M. House, and marveled at the changes wrought as by comparison with former methods. From my observation, wherever these methods are taught, the simplicity of the technic has been most advantageous.

There is a widespread desire among the members of the dental profession for advancement in the quality of their work, and for a better rendition of service to humanity.

In my opinion the one-week demonstrative courses would have been rendered popular because the dentists have felt that they could neither afford the sacrifice of time nor money for anything more exhaustive, but, in my opinion, these courses necessarily fall far short of actual teaching courses in actual value.

My own experience confirms my view that the only way is to do the work with my own hands under the guidance of a competent instructor and demonstrator. Such men are few and far between. Teachers are born, not made. Covering the standpoint of difference between teaching something, and then being able to actually perform what they teach—"there's the rub," and there's a wide chasm between.

My first experience in what may be called the new school of teaching for dentists who are in practice was when I had the opportunity of observing a course of one month under the auspices of Extension Division of the University of Minnesota, under the direction of Dr. M. M. House, of Indiana, in September, 1919, on full denture construction. The simplicity and teachableness of the technic was most noticeable and appealing and it was really marvelous how the men grasped the technic, and were able to produce with their own hands, under proper guidance, dentures that were wonderful from the standpoint of efficiency and esthetics.

The first week of the course was devoted to lectures, and the carrying through of one full upper and lower denture for an actual patient, and every phase of the work was executed before the class by Dr. House.

The second week of the course was in technic work, taking impressions, and bites from upper and lower casts mounted on Hall Articulators, this work being performed by the members of the class under the guidance of Dr. House and his assistants. The importance of this week of denture work by the class, and the construction of full upper and lower dentures following each detail of the technic throughout can not be overestimated. The class received a real insight into the simplicity, accuracy, and efficiency of the technic such as would be impossible otherwise to obtain.

The third and fourth weeks were devoted to the building of full dentures for actual patients furnished by the University. There were forty-six men in the class, two men to a patient, and they performed each step of the technic as taught, beginning with mouth examination and classification, impression taking, cast making, trial plates, taking the bite, mounting cases on articulator, articulating the teeth—everything complete to the finished denture.

Finally the class was taught how to make corrections for inaccuracies, which is just as essential as to know how to construct the dentures.

The last half day of the course was devoted to a general clinic in which every patient was present with the completed dentures in the mouth. The dentists of St. Paul and Minneapolis were invited to this clinic, and came in large numbers. I do not think these men will soon forget what their eyes were permitted to observe on that day, and they surely marveled at the results. The work as given proved to be so beneficial to all concerned that I understand similar courses are to be repeated this year.

In conclusion, I wish to say that intensive courses in our educational institutions and study clubs, properly conducted over a period of years in a practical way, mean the raising of standards, quality of service, and health to humanity, while to all practitioners who avail themselves of such opportunities it means inspiration, pleasurable occupation, good fellowship, and a truly successful professional career.—*Dental Digest.*

